# Investigation of the mechanisms of *Angelica dahurica* root extract-induced vasorelaxation in isolated rat aortic rings

**DOI:** 10.1186/s12906-015-0889-8

**Published:** 2015-10-31

**Authors:** Kyungjin Lee, Min Sik Shin, Inhye Ham, Ho-Young Choi

**Affiliations:** Department of Herbology, College of Korean Medicine, Kyung Hee University, 26 Kyungheedae-ro, Dongdaemun-gu, Seoul, 130-701 Republic of Korea

**Keywords:** *Angelica dahurica*, vasorelaxant effect, receptor-operated calcium channel, voltage-dependent calcium channel, hypertension

## Abstract

**Background:**

The root of *Angelica dahurica* Bentham et Hooker (Umbelliferae) has been used as a traditional medicine for colds, headache, dizziness, toothache, supraorbital pain, nasal congestion, acne, ulcer, carbuncle, and rheumatism in China, Japan, and Korea. Interestingly, it has been used in the treatment of vascular diseases including hypertension. The aim of this study was to provide pharmacological evidence for the anti-hypertensive effect of *A. dahurica* by investigating the mechanism underlying its vasorelaxant effect.

**Methods:**

The vasorelaxant effects of a 70 % methanol extract of the *A. dahurica* root (ADE) on rat thoracic aorta and its underlying mechanisms were assessed. Isolated rat aortic rings were suspended in organ chambers containing 10 ml Krebs-Henseleit (K-H) solution and placed between 2 tungsten stirrups and connected to an isometric force transducer. Changes in tension were recorded via isometric transducers connected to a data acquisition system.

**Results:**

ADE causes concentration-dependent relaxation in both endothelium-intact and endothelium-denuded aortic rings precontracted with phenylephrine (PE; 1 μM) or potassium (KCl; 60 mM) in K-H solution. And pre-treatment with ADE (1 mg/ml) inhibited calcium-induced vasocontraction of aortic rings induced by PE or KCl. However, ADE pre-treatment did not affect the contraction induced by PE or caffeine in Ca^2+^-free K-H solution.

**Conclusions:**

These results suggested that the ADE has vasorelaxant effect and the vasorelaxant activity is mediated by endothelium-independent pathway that includes the blockade of extracellular calcium influx through the receptor-operated Ca^2+^ channel and voltage-dependent calcium channel pathways.

## Background

*Angelica dahurica* Bentham et Hooker (Umbelliferae) is a perennial plant that grows widely in East Asia. The roots of this plant are used as a traditional medicine for colds, headache, dizziness, toothache, supraorbital pain, nasal congestion, acne, ulcer, carbuncle, and rheumatism [1, 2]. To date, the root has been reported to have antibacterial properties [3], wound healing effect [4], acetylcholinesterase inhibitory effect [5], anti-asthmatic effect [6], anti-staphylococcal effect [7], anti-acne effect [8], anti-inflammatory effect [9, 10], antitumor effects [11], protective effect against sepsis [12], and hypotensive properties [13]. Furthermore, imperatorin isolated from root of *A. dahurica* showed anti-cancer effect [6], anti-oxidant effect [14], hepatoprotective effect [15] and vasodilation activity [16].

Several previous studies have focused on the vascular activities of *A. dahurica* due to the frequent use of the herb, together with other herbs in clinical use, to relieve stagnant Qi, promote blood flow, and treat blood stasis [13, 14, 17–19]. Furthermore, imperatorin, one of major component of *A. dahurica*, is reported to have anti-hypertensive and vascular remodeling effects [20, 21], and vasodilation activity [22].

Recently there has been increased interest in the hypotensive effects of traditional medicines which have been used in China, Japan and Korea for several thousand years [23–26].

Therefore, the purpose of the current study was to provide pharmacologic evidence for the traditional use of *A. dahurica* in the treatment of vascular diseases by determining the probable mechanisms involved in its vasorelaxant effect.

## Methods

### Chemicals and drugs

Phenylephrine hydrochloride (PE), acetylcholine (Ach), ethylene glycol-bis(β-aminoethyl ether)-N,N,N′,N′-tetraacetic acid (EGTA), potassium chloride (KCl), calcium chloride (CaCl_2_), and caffeine were purchased from Sigma Aldrich (St Louis, MO, USA). Barium chloride was purchased from Wako Pure Chemical Industries (Osaka, Japan). All other reagents were of analytical purity.

### Plant material and extraction

*A. dahurica* was collected from Uiseong, Gyeongbook Province, Republic of Korea in 2008. Plant identification was performed by Professor Hocheol Kim of Kyung Hee University, Seoul, Republic of Korea. A voucher specimen (AD001) of *A. dahurica* was deposited at the College of Korean Medicine, Kyung Hee University. A crude extract was prepared by decoction of dried root and rhizome of A. dahurica (3.0 kg) in methanol (3 L) for 3 times (120 min per time). After reflux and filtration, the extract was evaporated using a rotary evaporator at 70 °C and lyophilized to yield 912.0 g of crude extract. The crude extract was dissolved in Krebs-Henseleit (K-H) solution when applying to aortic rings in organ chamber.

### High performance liquid chromatography (HPLC) analysis

HPLC analysis was performed at room temperature at a flow rate of 1.0 ml/min by a Gilson system equipped with a 234 autosampler, a UV/VIS-155 detector, and a 321 HPLC Pump (Gilson, Seoul, Korea). The injection volume was 2 μl. A LUNA 4.60 × 250 mm C_18_ reverse-phase column with 5-μm particles (Phenomenex, CA, USA) was used. The mobile phase consisted of acetonitrile (A) and distilled water (B) (HPLC grade, J. T. Baker Co. LTD., USA) at the ratio of 6:4. The column eluent was monitored at 254 and 365 nm.

### Animals

All animal procedures were conducted according to the animal welfare guidelines issued by the Kyung Hee University Institutional Animal Care and Use Committee (KHUASP[SE]-09-042). Male Sprague–Dawley rats (*N* = 30, Narabio, Seoul, Republic of Korea) weighing 240–260 g were housed under controlled conditions (temperature, 22 ± 2 °C; lighting, 07:00–19:00 h), with food and water available *ad libitum*.

### Experimental protocols

The method of preparation of rat aortic rings has been described previously [27]. Isolated rat aortic rings were suspended in organ chambers containing 10 ml K-H solution and placed between 2 tungsten stirrups and connected to an isometric force transducer. Changes in tension were recorded via isometric force transducers connected to a data acquisition system. Effects of a 70 % methanol extract of the *A. dahurica* root (ADE) on PE- and KCl-induced contraction: Endothelium-intact aortic rings were precontracted by PE (1 μM) or KCl (60 mM) in standard Krebs-Henseleit (K-H) solution. After a plateau was reached, cumulative doses (0.03–3.0 mg/ml) of ADE were added. The relaxant effect on the aortic rings was expressed as a percentage of contraction induced by PE or KCl.

Role of endothelium in ADE-induced relaxation: The concentration-dependent relaxant effect of ADE was studied in endothelium-intact and endothelium-denuded aortic rings precontracted by PE (1 μM) in standard K-H solution. After contraction with PE, Ach (10 μM) was added in order to confirm the absence or presence of endothelium. After washing, rings were contracted with PE (1 μM). After contraction with PE, cumulative doses (0.03–3.0 mg/ml) of ADE were added. In the previous screening study, ADE did not relax PE-precontracted aortic rings at dose of 0.001, 0.003, and 0.01 mg/ml. Therefore, vasorelaxant effects of ADE was observed at the dose of 0.03 mg/ml. The relaxant effect on the aortic rings was expressed as a percentage of the contraction induced by PE.

Effects of ADE on extracellular Ca^2+^-induced contraction: The vasorelaxant activities of ADE (1 mg/ml) on the receptor-operated Ca^2+^ channel (ROCC) and voltage-dependent calcium channel (VDCC) in Ca^2+^-free K-H solution were investigated in the same manner as we previously described [27].

Effects of ADE on intracellular Ca^2+^ release: In order to investigate the effects of ADE on intracellular Ca^2+^ release from sarcoplasmic reticulum (SR)-induced contraction via specific inositol triphosphate receptor (IP_3_R) channels or ryanodine receptor (RyR) channels, the contractile activities of PE (1 μM) or caffeine (5 mM) were investigated on endothelium-denuded aortic rings after 10 min of pretreatment with ADE (1 mg/ml) in Ca^2+^-free K-H solution.

### Data analysis

Data were expressed as mean ± standard error of mean (SEM). Statistical comparisons were made using Student’s *t*-test. All statistical analyses were performed using SPSS (version 10.0) statistical analysis software (SPSS Inc., Chicago, IL, USA). *P* values less than 0.05 were considered to be statistically significant.

## Results

### Vasorelaxant effects of ADE

**Fig. 1:**
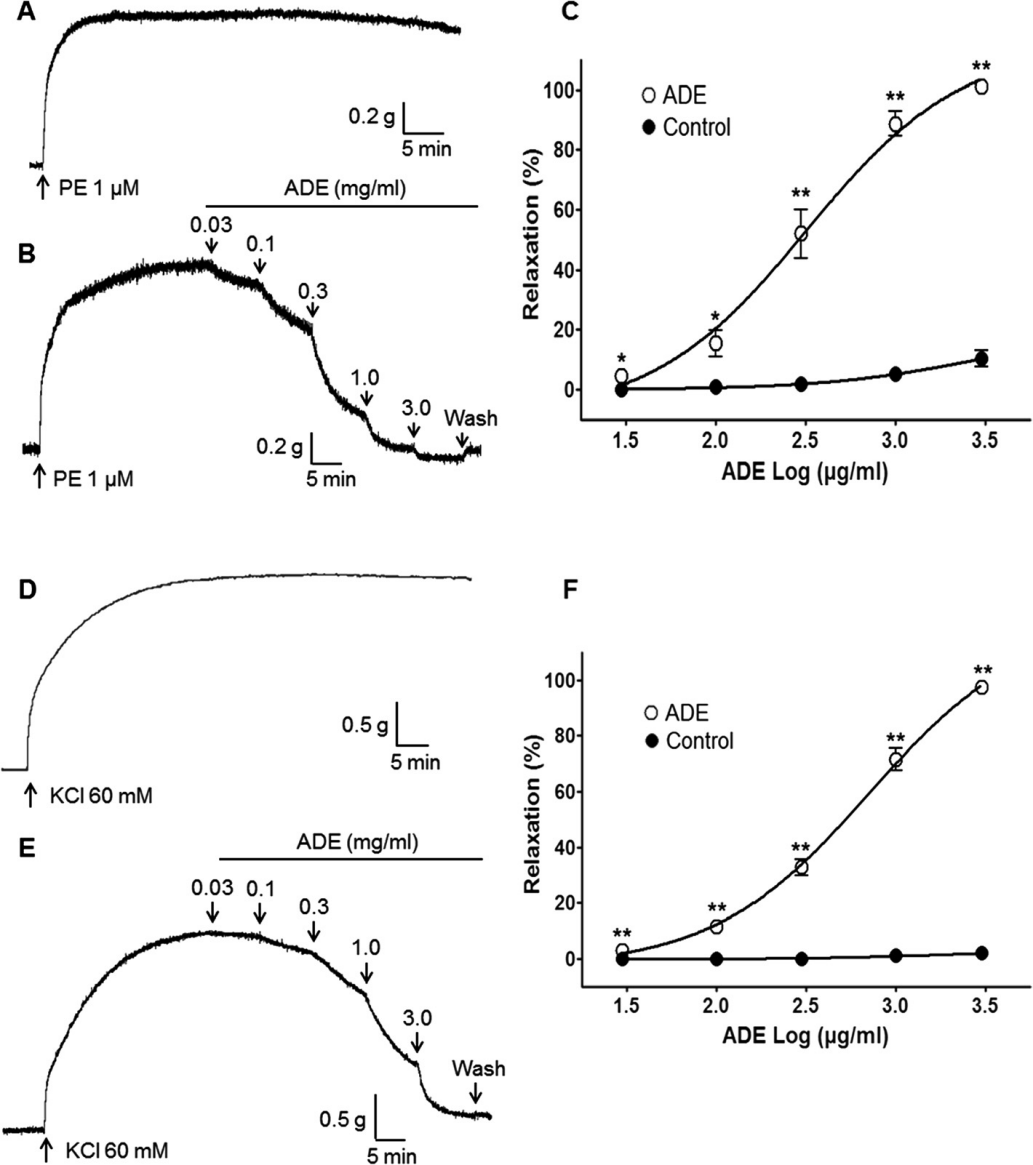
Vasorelaxant effect of a 70 % methanol extract of *Angelica dahurica* root (ADE) on phenylephrine (PE)-precontracted (**a**, **b**, and **c**) or KCl-precontracted (**d**, **e**, and **f**) rat aortic rings. Representative traces show the control group (**a** and **d**) and the ADE-treated group (**b** and **e**). Control groups were treated with the same volume of Krebs-Henseleit solution without ADE. Values are expressed as mean ± SEM (*n* = 6–8). ^*^ *P* < 0.05, ^**^ *P* < 0.01 vs. control

ADE (0.03–3.0 mg/ml) relaxed PE-precontracted (1 μM) endothelium-intact aortic rings in a concentration-dependent manner (Fig. 1a, b and c). ADE (0.03–1.0 mg/ml) also relaxed KCl-precontracted (60 mM) endothelium-intact aortic rings in a concentration-dependent manner (Fig. 1d, e and f).

### Role of endothelium in ADE-induced relaxation

**Fig. 2:**
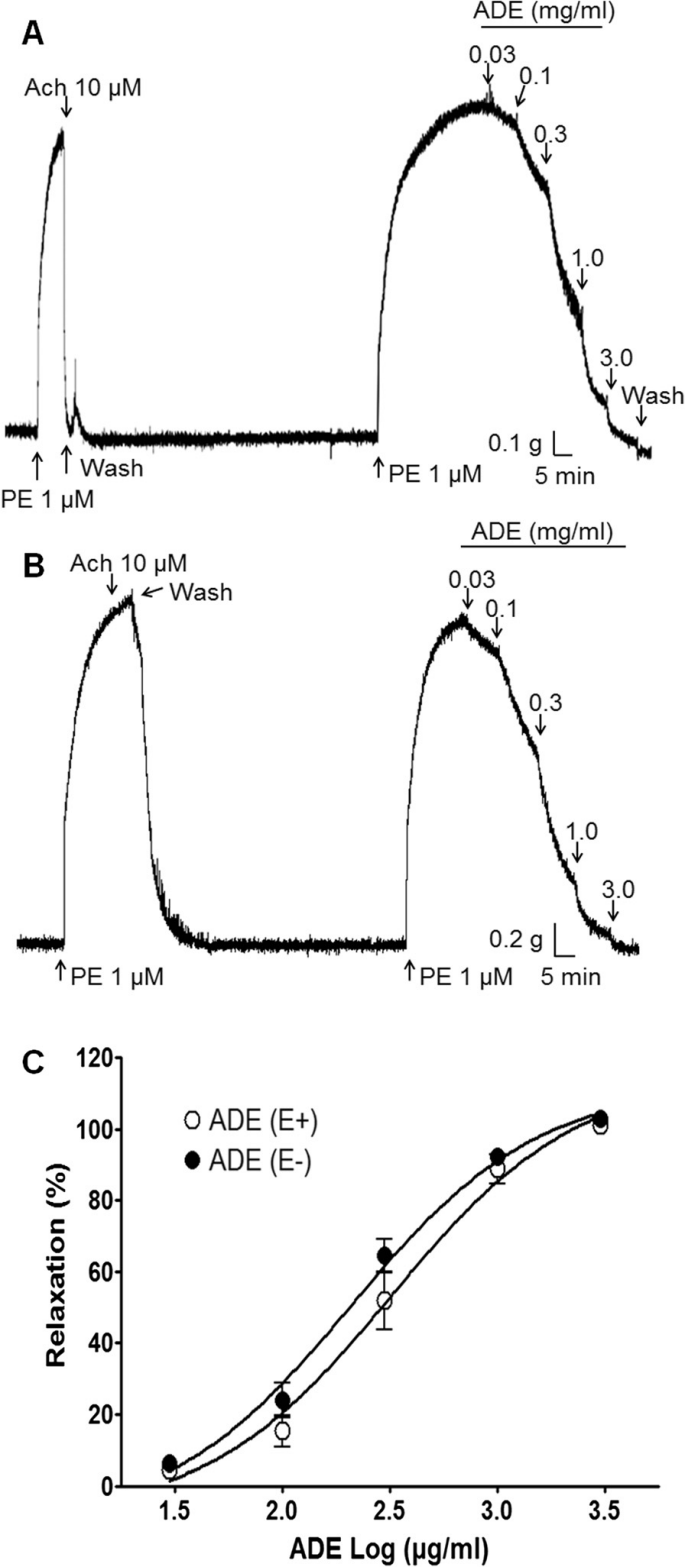
Vasorelaxant effect of a 70 % methanol extract of *Angelica dahurica* root (ADE) on phenylephrine (PE)-precontracted (1 μM) rat aortic rings with (E+) (**a**) or without (E-) (**b**) endothelium. PE-induced contraction of endothelium-intact aortic ring almost relaxed to baseline after acetylcholine treatment (Ach, 10 μM) (**a**). PE-induced contraction of endothelium-denuded aortic ring remained unaffected after acetylcholine treatment (**b**). Values are expressed as mean ± SEM (*n* = 8) (**c**)

ADE showed a concentration-dependent relaxation effect in both endothelium-intact and endothelium-denuded aortic rings after precontraction by PE (1 μM). However, the functional removal of endothelium did not modify ADE-induced relaxation in PE-precontracted rat thoracic aorta rings (Fig. 2).

### Effects of ADE on Extracellular Ca^2+^-induced Contraction

**Fig. 3:**
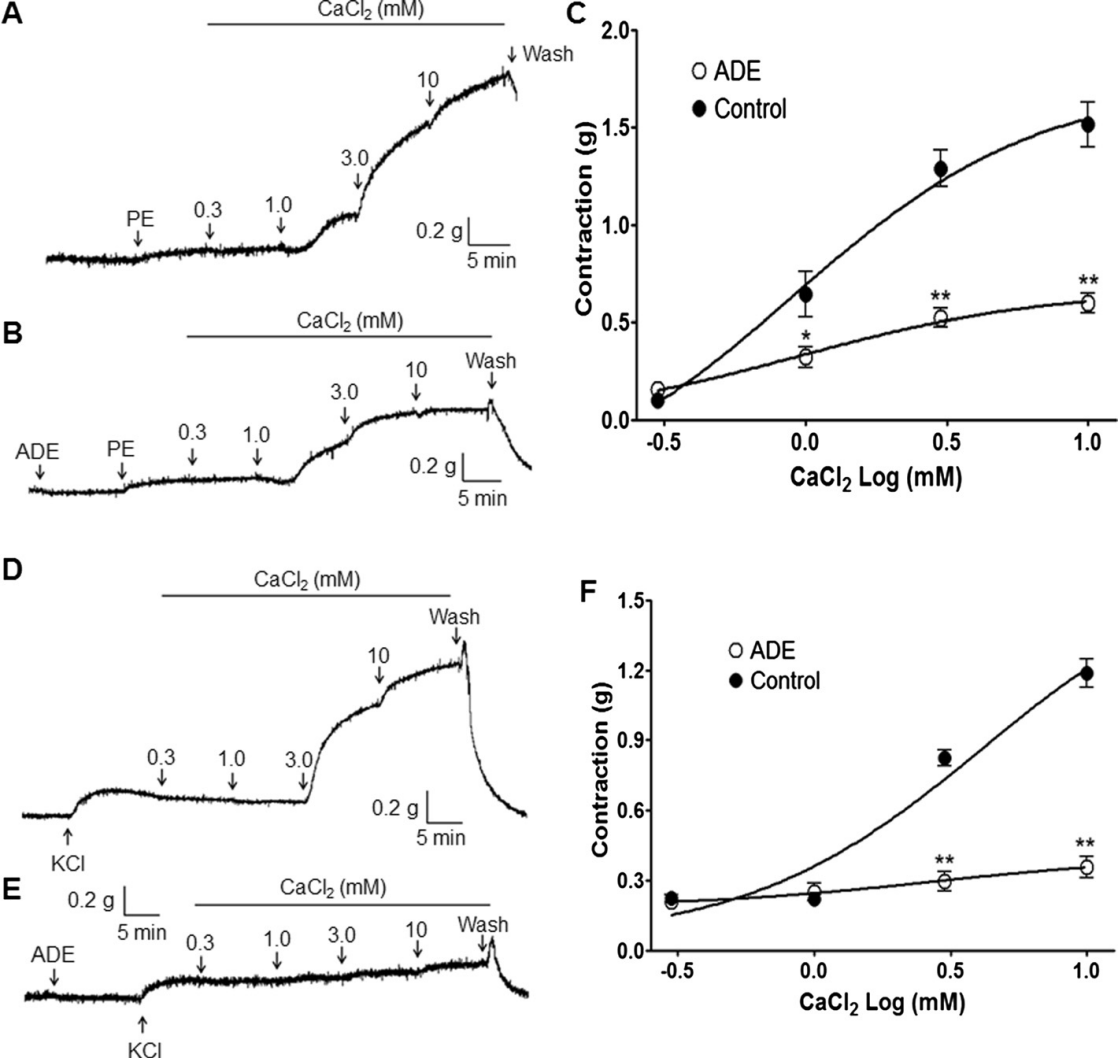
Inhibitory effects of a 70 % methanol extract of *A. dahurica* root (ADE, 1 mg/ml) on extracellular Ca^2+^-induced (0.3–10 mM) contraction in endothelium-denuded rat aortic rings precontracted by phenylephrine (PE, 1 μM) (**a**, **b**, and **c**) or KCl (60 mM) (**d**, **e**, and **f**) in Ca^2+^-free solution. Representative traces show the control group (**a** and **d**) and the ADE-treated group (**b** and **e**). Control groups were not pre-incubated with ADE. Values are expressed as mean ± SEM (*n* = 4–8). ^*^ *P* < 0.01, ^**^ *P* < 0.01 vs. control

In order to investigate the effects of ADE on the ROCC and VDCC pathways, PE (1 μM) and KCl (60 mM) were applied to induce stable contraction, respectively. Pre-treatment with ADE (1 mg/ml) significantly inhibited the contraction induced by extracellular CaCl_2_ (0.3–10 mM) compared to the control group (Fig. 3).

### Effect of ADE on SR calcium release induced by PE or caffeine

**Fig. 4:**
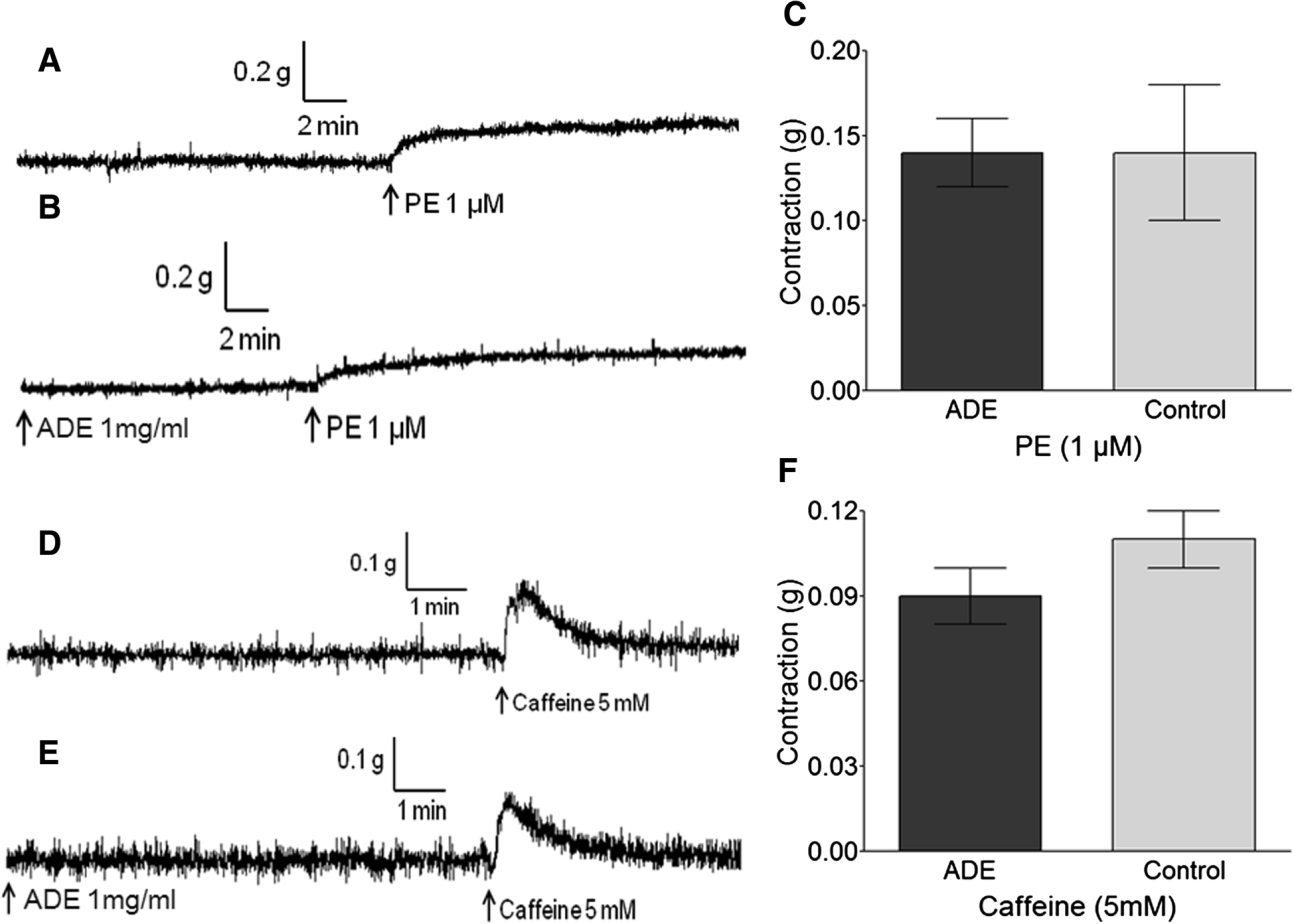
Inhibitory effect of a 70 % methanol extract of *A. dahurica* root (ADE, 1 mg/ml) on contraction via SR calcium release (the IP_3_ pathway) by PE (1 μM) in Ca^2+^-free solution (**a**, **b**, and **c**) and on contraction via sarcoplasmic reticulum calcium release (the RyR pathway) by caffeine (5 mM) (**d**, **e**, and **f**) in Ca^2+^-free solution. Representative traces show the control group (**a** and **d**) and the ADE-treated group (**b** and **e**). Control groups were not pre-incubated with ADE. Values are expressed as mean ± SEM (*n* = 8)

In Ca^2+^-free K-H solution, pre-treatment with ADE (1 mg/ml) for 10 min did not inhibit PE-induced (1 μM) contraction or caffeine-induced (5 mM) contraction (Fig. 4).

### HPLC analysis of ADE

**Fig. 5:**
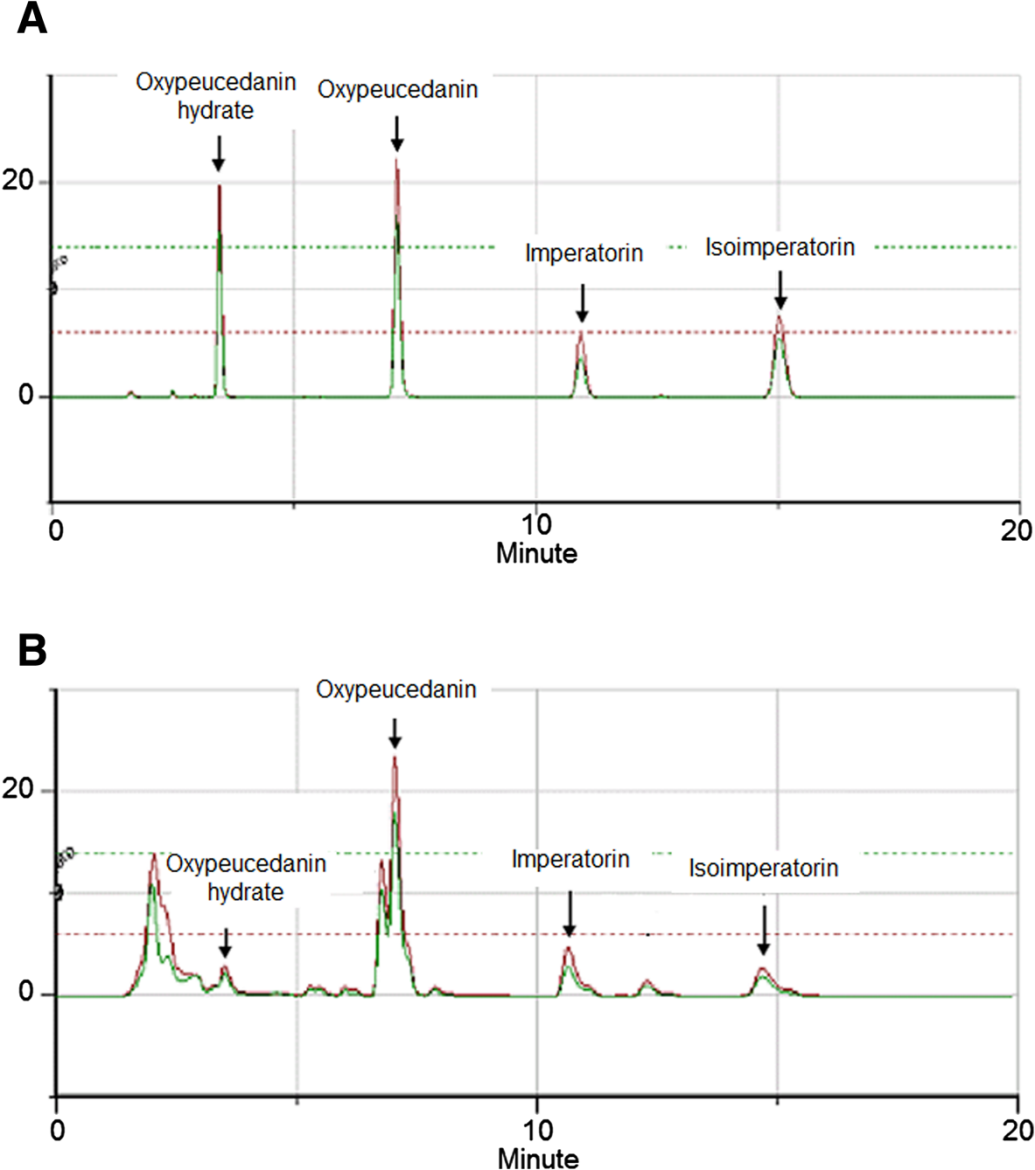
High performance liquid chromatography analysis of standard mixture (**a**) and a 70 % methanol extract of *A. dahurica* root (**b**). Green lines come from 254 nm and red lines come from 365 nm

Four standard components of *A. dahurica*, oxypeucedanin hydrate, oxypeucedanin, imperatorin, and isoimperatorin were found in ADE (Fig. 5). And the contents of oxypeucedanin hydrate, oxypecedanin, imperatorin, and isoimperatorin in ADE were calculated to 0.23, 5.9, 0.63, 0.43 %, respectively.

## Discussion

Vascular tone is the major factor determining the blood flow through the circulatory system. Regulation of the vasoactivity of vascular smooth muscle is dependent on a complex interplay of vasodilatory and vasoconstrictory actions by circulating hormones, neurotransmitters, and endothelium-derived factors [28]. The vasorelaxant effect is usually classified as endothelium dependent or independent depending on endothelial function. The endothelium regulates vascular smooth muscle tone through the secretion of vasorelaxant substances such as nitric oxide, prostacyclin, and endothelium-derived hyperpolarizing factor [29], as well as through endothelium-derived contracting factors such as endothelins, angiotensin II, cyclooxygenase-derived prostanoids, and superoxide anions [30]. In the present study, ADE evoked a concentration-dependent relaxation of aortic rings precontracted by the application of PE (1 μM) or KCl (60 mM). The removal of functional endothelium did not change these responses. These results indicated that the vasorelaxant mechanism of ADE may not be regulated by endothelium-dependent factors.

Vascular smooth muscle contraction generally occurs through pharmacomechanical or electromechanical coupling [31]. Pharmacomechanical coupling involves the activation of cell-surface receptors, such as the α-adrenergic receptor, to increase extracellular Ca^2+^ influx through the ROCC or the release of Ca^2+^ from intracellular stores. In contrast, electromechanical coupling involves the depolarization of the cell membrane to increase extracellular Ca^2+^ influx through the VDCC [32]. PE, an alpha-adrenergic agonist, contracts smooth muscle cells through the extracellular Ca^2+^ influx in ROCCs and through the release of internal Ca^2+^ from specific IP_3_R channels in the SR membrane [33, 34]. KCl contracts smooth muscle cells mainly by the extracellular Ca^2+^ influx that occurs with the depolarization of the cell membrane and subsequent opening of VDCCs [35]. Caffeine contracts smooth muscle cells by internal calcium release from RyR channels in the SR membrane [36]. Therefore, the vasorelaxant effects of ADE were investigated using vasoconstrictors such as PE, KCl, and caffeine on the following physiologic processes: extracellular Ca^2+^ influx via the ROCC or VDCC pathways and intracellular Ca^2+^ release via IP_3_R or RyR channels.

In the present study, ADE (1 mg/ml) inhibited the vasocontraction induced by Ca^2+^ supplementation in rat aortic rings that were precontracted with PE (1 μM) in a Ca^2+^-free K-H solution. This result suggests that ADE can inhibit the vasocontraction induced by extracellular Ca^2+^ entry via the ROCC pathway. And ADE (1 mg/ml) also inhibited the vasocontraction induced by Ca^2+^ supplementation in the aortic rings precontracted with KCl (60 mM) in a Ca^2+^-free K-H solution. This result suggests that ADE may also inhibit the vasocontraction induced by extracellular Ca^2+^ entry via the VDCC pathway.

In a Ca^2+^ free K-H solution, PE-induced contraction is only mediated by the IP_3_ pathway [33]. In the present study, a 10-min pretreatment with ADE (1 mg/ml) did not inhibit PE-induced (1 μM) contraction. This result suggests that ADE-evoked vasorelaxation may not be related to internal calcium release from specific IP_3_R channels in the SR.

In the present study, a 10-min pretreatment with ADE (1 mg/ml) did not inhibit caffeine-induced (5 mM) vasoconstriction. This result suggests that ADE-evoked vasorelaxation also does not affect internal calcium release from RyR channels in the SR.

In this study, 4–8 rat aortic rings were used in the control and experimental groups. This number of aortic rings could be considered not statistically significant because 4 more aortic rings could be isolated from one rat. In the present study, we isolated 4 aortic rings from one rat and used 2 rings as the control group and 2 rings as the experimental group. In Fig. 3, the control group comprised of 4 rings, while the ADE-treated group comprised of 8. In other words, 2 rats were used as the control group and 3 rats were used in the experimental group. We conducted various experiments and found that the contractions in the control group were not different from our previous experiments and there are little variations. Therefore, we used only 2 rats (4 rings) in the control group. In the *in vivo* experiment, many variations were observed in individual animals. However, in *ex vivo* experiments using aortic rings, there are little variations in individual animals. In Korea, minimum use of animals is recommended for animal welfare. We agree with your comments that more number of rings could be statistically significant. However, our experiments are *ex vivo* experiments using isolated rat aortic rings, and we believe that our results have statistical significance.

Oxypeucedanin, oxypeucedanin hydrate, imperatorin, and isoimperatorin are well known components of *A. dahurica* root [37–40]. In the present study, these four components were also found in ADE and the contents were calculated as 5.9, 0.23, 0.63, and 0.43 %, respectively. Imperatorin is a well-known anti-hypertensive compound [20–22]. In addition, Bertin et al. showed that atropine and L-N^G^-nitro-arginine methyl ester did not alter imperatorin-induced relaxation in rat aorta [21]. In the present study, ADE also relaxed the rat aortic rings pre-contracted with PE or KCl in an endothelium-independent manner. Therefore, the vasorelaxant effects of ADE might be partly attributable to imperatorin. However, Nie et al. suggested that imperatorin relaxed the mouse thoracic aorta pre-contracted with PE in an endothelium dependent manner [16]. These different results might be attributed to the use of mouse thoracic aorta. Although oxypeucedanin was the most abundant component in ADE, its vasorelaxant or anti-hypertensive activities have not been reported yet. Oxypeucedanin hydrate and isoimperatorin also have not been reported to have vasorelaxant activities or anti-hypertensive activities. Therefore, further phytochemical and pharmacological studies of ADE are needed.

## Conclusion

In conclusion, our results suggest that ADE has vasorelaxant activity and the vasorelaxant activity of ADE is mediated by endothelium-independent pathway that includes the blockade of extracellular calcium influx through the ROCC and VDCC pathways. In addition, intracellular calcium-release pathways through the IP_3_R and RyR channels were not involved in the vasorelaxant effect of ADE.

## Statement of ethics

This study was submitted to, and approved by the Kyung Hee University Institutional Animal Care and Use Committee (Approval number: KHUASP[SE]-09-042).
